# St. Andrew's Hospital, Northampton

**Published:** 1906-10-13

**Authors:** 


### ST. ANDREW'S HOSPITAL, NORTHAMPTON.

This institution is one of the most complete private establishments for the reception of mental patients in the kingdom. It is intended for patients of the middle and upper classes. The general accommodation is all that could be desired, and besides this there are prviate suites of rooms for those who prefer them. The hospital is situated within easy distance from London, one mile from Northampton. The establishment consists of a fine principal building at Northampton, a sea-side house in North Wales, and villas in the hospital grounds, and also at Moulton Park House, two miles away from St. Andrews. The interior of St. Andrew's Hospital is beautifully furnished and arranged, and it is not surprising that the inmates are contented with the arrangements. There are fine billiard-rooms and comfortable, spacious, light and airy corridors and sitting-rooms. The theatre and recreation-room is a fine apartment, and is handsomely re-decorated. A delightful little chapel stands in the grounds. Here, too, good provision has been made for numerous sports. The bowling-green is surrounded by shrubs and trees, there is a good croquet-lawn, lawn tennis-ground, and full-sized cricket ground. In the carriages of the establishments those inmates who are sufficiently well take frequent drives, whilst the richer patients may hire carriages and horses or keep their own. A servant, if required, is accommodated in the suites for patients. The seaside home, Bryn-y-Neuadd Hall, is most beautifully situated on a wooded slope, and the public apartments and appointments here are quite magnificent. The ball-room is in the style of a baronial hall, with a fine ceiling and elaborately carved furniture. The grounds and the garden are in keeping with the interior. These grounds consist of 280 acres, and those of St. Andrew's Hospital are of 200 acres in extent. At this beautiful establishment, the charges are from one and a half to four guineas per week. Patients requiring special apartments and privileges are charged from £8 per week. Lord Spencer is President of St. Andrew's Hospital.

